# Saffron and its active ingredients against human disorders: A literature review on existing clinical evidence

**DOI:** 10.22038/IJBMS.2022.63378.13985

**Published:** 2022-08

**Authors:** Seyedeh Farzaneh Omidkhoda, Hossein Hosseinzadeh

**Affiliations:** 1 Pharmaceutical Research Center, Pharmaceutical Technology Institute, Mashhad University of Medical Sciences, Mashhad, Iran; 2 Department of Pharmacodynamics and Toxicology, School of Pharmacy, Mashhad University of Medical Sciences, Mashhad, Iran

**Keywords:** Clinical trial, Crocin, Disease, Disorder, Saffron, Therapy

## Abstract

Saffron, the stigmas of Crocus sativus L., has been mentioned extensively in the traditional reference texts as a herbal medicine. Many clinical trials have been conducted on this valuable herbal substance and its main constituents following numerous cellular and animal assessments. In the present review, we have collected almost all of these clinical studies to clarify how much knowledge has clinically been achieved in this field so far and which scientific gaps are needed to be filled by more studies. A comprehensive literature review was conducted through a two-round search. First, we performed a general search for identifying the human disorders against which saffron was studied. Then, we searched specifically for the combination of saffron keywords and each disease name. Scientific databases including Scopus, PubMed, and Web of science were used for this search. Studies were collected through electronic databases from their inception up to August 2021. The largest number of these clinical studies represent the investigations into saffron efficacy in different neurological and mental disorders, particularly depression. This substance has clinically revealed significant protective effects against various types of depression, age-related macular degeneration, and allergic asthma. In some cases, such as sexual dysfunction, cognitive and metabolic disorder, the effects of saffron are still clinically open to dispute, or there are limited data on its positive influences. Overall, saffron and its constituents have promising effects on human disorders; however, it needs more clinical evidence or meta-analyses to be confirmed.

## Introduction

Nature is the best innovator to introduce novel therapeutic agents or at least novel precursors for semi-synthetic drugs. Logically, natural compounds are more likely to be biologically compatible with the human body than those chemically synthesized because firstly, they are produced by other living organisms, and secondly, they have probably been tested, used, and then introduced to us by our ancestors, generation by generation. Furthermore, now a lot of effective natural drugs or natural substance-derived drugs, such as some antibiotics or anticancer medications have been developed and clinically been applying (1). Thus, natural, abundant resources should be carefully screened.

Saffron, stigmas of *Crocus sativus* L. plant, has been mentioned extensively in the traditional reference texts as a herbal medicine. It is also widely used as food coloring and flavoring (2). The role of saffron in the food industry not only has been preserved but also developed so far (3). Moreover, in modern pharmacological studies, the effects of saffron and its most important constituents, crocin, safranal, crocetin, and picrocrocin against neurological, behavioral, cardiovascular, ocular, and metabolic disorders have been investigated especially in animal models. There are also a lot of clinical studies showing the beneficial effects of saffron or its active ingredients as the main or complementary treatment regimen in mentioned diseases (4-7). These promising effects of this valuable substance have resulted in developing some patent products or producing some pharmaceutical formulations available in drug stores as supplements in some countries (8).

**Figure 1:**
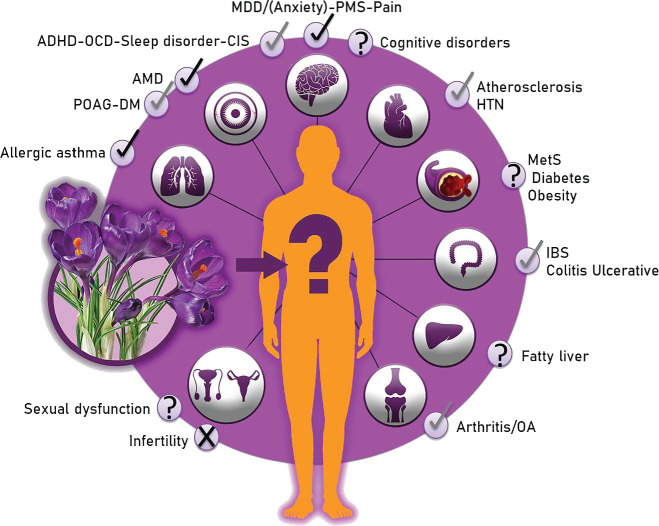
Effects of saffron and its constituents were assessed on different human diseases. The question mark shows controversial effects, the black tick mark shows significant effects, the gray tick mark shows relatively significant effects and the multiplication sign shows no significant effects

MDD: Major depressive disorder; PMS: Premenstrual syndrome; HTN: Hypertension; MetS: Metabolic syndrome; IBS: Irritable bowel syndrome; OA: Osteoarthritis; ADHD: Attention deficit hyperactivity disorder; OCD: Obsessive-compulsive disorder; CIS: Cerebral ischemic stroke; AMD: Age-related macular degeneration; POAG: Primary open-angle glaucoma; DM: Diabetic maculopathy

In this comprehensive review, we will discuss the clinical trials which have been performed to investigate the protective effects of saffron against different human disorders (Figure 1) in separate parts.

## Method

**Figure 2:**
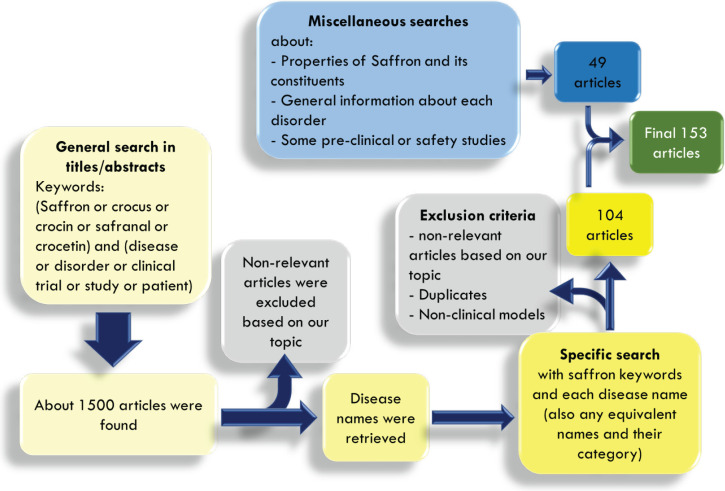
Flowchart of the search process and exclusion criteria

**Figure 3:**
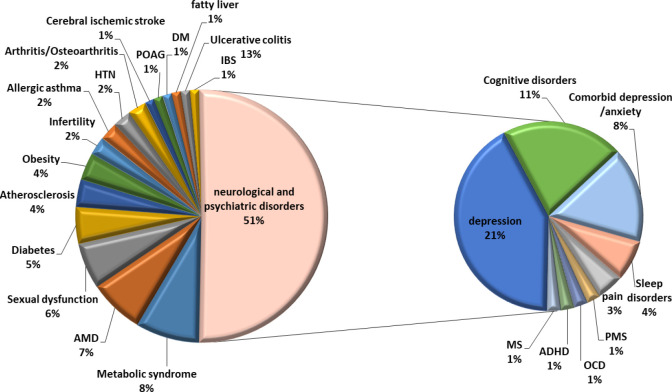
Percentage of clinical trials in each disorder

PMS: Premenstrual syndrome; OCD: Obsessive-compulsive disorder; ADHD: Attention deficit hyperactivity disorder; AMD: Age-related macular degeneration; POAG: Primary open-angle glaucoma; DM: Diabetic maculopathy; HTN: Hypertension; IBS: Irritable bowel syndrome; MS: Multiple sclerosis

A comprehensive literature review was conducted through a two-round search. In the first round, general related keywords were chosen to make the search results broad enough, including (saffron or crocin or safranal or crocetin or crocus) and (disease or disorder or clinical or trial or study or patient) with the title/abstract filter. Then, the most relevant articles were retrieved and the second search was specifically performed using the above-mentioned saffron keywords in combination with each of obtained disease names, their equivalents, and derivatives (for example “major depressive disorder” or depression or depressive). Scientific databases including Scopus, PubMed, and Web of science were used for searching. Studies were collected through electronic databases from their inception up to August 2021. The advanced search process was conducted only among human clinical trials (details in Figure 2). Figure 1 has been prepared by Adobe Photoshop 2020, Figure 2 by Microsoft Powerpoint 2010, and Figure 3 by Microsoft Excel 2010. 

***Neurological and psychiatric disorders***

The largest number of clinical studies on saffron belongs to this part which consists of a broad spectrum of diseases listed below and discussed in detail, including Alzheimer’s disease (AD), depression, anxiety, premenstrual syndrome (PMS), obsessive-compulsive disorder (OCD), attention deficit hyperactivity disorder (ADHD), sleep disorder, pain, ischemic stroke, and multiple sclerosis (MS) (Figure 2). It is interesting to know that the effects of saffron and its constituents on neurological disorders are not limited only to the mentioned cases and there are also “preclinical” studies evaluating the effects of saffron on MS (9, 10), Parkinson’s disease (11, 12), Post-traumatic stress disorder (PTSD) (13), neuropathic pains (14, 15), schizophrenia (16), spinal cord injury (17, 18), tremor (19), traumatic brain injury (20), and cerebral ischemia (21, 22), but they have not reached the clinical phase yet. 

***Alzheimer’s disease (AD)***

Alzheimer’s disease is typically accompanied by memory and cognition decline, dependency on others, experiencing psychological complications such as hallucination in some cases, and early death. AD has a social and economic burden on communities and it is predicted to be 3 times more prevalent in 2050, because of higher population ages in the future. At present, although there is no definite cure for AD, pharmacotherapy with Acetyl-cholinesterase inhibitors including donepezil, galantamine, rivastigmine, and N-methyl-D-aspartate (NMDA) receptor antagonist, memantine, can help patients to have milder symptoms. Some non-pharmacological treatments can also relatively improve their quality of life (23).

Up to now, nine clinical trials have been conducted to assay the effects of saffron on different levels of neurocognitive impairments. The first study was performed from 2007 to 2009 and it was multicenter, randomized, and double-blind. In this study, two groups of 27 patients with mild-to-moderate AD were given only either saffron (15 mg/d during the first month and then 30 mg/d to the end) or donepezil (5 mg/d during the first month and then 10 mg/d to the end) for 22 weeks. The findings showed that the effects of total ethanol extract of saffron on global cognitive and clinical signs and symptoms of patients (monitored by Alzheimer’s Disease Assessment Scale-cognitive subscale (ADAS-cog) and Clinical Dementia Rating Scale-Sums of Boxes (CDR-SB)) were comparable to the effects of donepezil with fewer side effect (vomiting). Although both groups of patients were improving in terms of the scales, neither donepezil nor saffron had statistically significant effects after 22 weeks in comparison with the baseline. The other point is that there was no placebo arm in this study to detect any placebo effects (24). The other similar study had a shorter duration (16 weeks) and fewer volunteers (46 cases), but it was controlled with a placebo. The results of this clinical trial demonstrated a significant positive effect of saffron capsules (15 mg, BID) on patients in comparison with those who received a placebo. The efficacy of saffron in this study, after shorter duration of treatment, was surprisingly more than in the aforementioned trial and unlike it, the cognitive profiles of cases were significantly better than the baseline in the saffron group (25). The alleviating effects of saffron on mild cognitive impairment were also demonstrated by another one-year clinical study; however, it has several serious weak points, the most important of which include not mentioning the amount of saffron administered, no placebo group, small sample size (35 subjects), and no randomized grouping (26). Cicero and his co-workers (2016) used saffron (30 mg) in combination with *Bacopa monnieri *dry extract (320 mg), L-teanina (100 mg), some vitamins (B_6 _(9.5 mg), B_7_ (450 mcg), B_9_ (400 mcg), B_12_ (33 mcg), D (25 mcg)) and copper (2 mg) in one capsule to assess its effects on self-perceived (not previously diagnosed) cognitive impairment. This human assessment was conducted on 30 participants for a month. Cognitive function including orientation, attention, memory, language, and visual-spatial skills of participants was evaluated by Mini-mental State Examination (MMSE) only at the beginning and the end of the study; therefore, the trend of alterations has not been determined. One of these nutraceutical capsules per day, after just one month, could significantly increase the MMSE score compared with the initial level and the randomized placebo group. Thus, it can be inferred that concurrent intake of these herbal extracts and vitamins could potentiate the final effectiveness (27). 

Two other clinical studies worked on moderate-to-severe or major cognitive disorders. Farokhnia and his colleagues revealed that 30 mg/day of saffron could diminish moderate to severe AD, which is statistically comparable with 20 mg/day of memantine. In this trial with 68 cases, finally, those who received saffron and memantine respectively had 9.18% and 7.79% decreases for the score of cognitive dysfunction (Severe Cognitive Impairment Rating Scale, SCIRS). In contrast, the related figure of the article reveals the average effect of memantine on SCIRS scores was a little more than saffron during the first 6 months of the study. Consequently, saffron possibly needs more time to be effective. It is also important to say that the statistical comparison of the final effectiveness of both treatments with their baseline has not been reported and also there is no placebo group in this study, so we cannot conclude the definite protective effect of saffron and even memantine against moderate to severe AD (28). The latest clinical study in 2018 has evaluated the effects of a mixed formulation containing saffron (30 mg) and sedge (500 mg) given twice a day, and Astragalus honey (5 g) given daily on patients with previously diagnosed major cognitive disorder. This herbal combination was administered as a supplement for patients who were taking their main anti-AD medications. The trial was randomized, double-blind, and placebo-controlled on 60 participants. The findings suggest significant beneficial effects of this 2-month complimentary treatment compared with the placebo-treated patients, in the aspects of attention, memory, language, and visual-spatial function; nevertheless, fluency of the test subjects was not significantly affected. The dosage of saffron used in this study was twice as much as what has been chosen in most studies. Considering that the participants were taking their main medications concurrently, the results would have been more reliable if they had set a similar main medication plan for all of them, if possible, or they had reported and compared their mediations in both groups. Another interfering factor in this study was that the education level in the test group was significantly higher than the control group, which may have affected the patients’ adherence to the therapies (29). 

The studies mentioned above have been performed on cases suffering from cognitive disorders without any unstable severe diseases. Moazen-Zadeh *et al*. (2017) studied the impact of saffron on cognitive decline developed following Coronary Artery Bypass Graft (CABG) surgery in 37 patients (in a randomized, double-blind, and placebo-controlled trial). Finally, the researchers concluded that the 3-month intervention with 30 mg/day of saffron (divided into two doses, started from two days before surgery) failed to be significantly effective on MMSE scores in comparison with the baseline and the placebo (30). Neurocognitive impairment as a neurological side effect of medical interventions was also considered by other researchers in 2012. Their purpose was to examine saffron (30 mg) in combination with *Cyperus rotundus* (500 mg) and honey (5 g), against electroconvulsive therapy (ECT)-induced decreased memory in a randomized and double-blind trial. Eighty-four candidates for ECT (3 times a week for 6–10 sessions) received this herbal combination twice a day, from the start of ECT, for 40 days. The cognitive score of these subjects was evaluated (using Addenbrooke’s Cognitive Examination-Revised (ACE-R)) before ECT and at the different stages after it. In the end, they realized that the ACE-R score in the patients treated with the herbal combination was significantly higher after the first and second months from the last session of ECT. However, contrary to the purpose of this trial, there is no significant memory decline in the placebo-treated group after ECT. Accordingly, we can solely conclude that these herbs have an improving effect on the cognition of these cases in a time-dependent manner (31). 

The positive influence of ethanol extract of *C. sativus* petals on visual short-term memory (VSM) of humans was demonstrated in another human study. In this study, performers firstly examined 20 subjects with three psychophysical tests presenting the VSM function (contrast sensitivity, retention of VSM, and *n*-back memory test), then treated them with either petal extract of *C. sativus* (30 mg/day) or placebo (10 subjects in each group) for 3 weeks and finally repeated the three examinations. Observations revealed that this extract significantly amplified human VSM, whereas cases in the placebo group were not remarkably affected (32).

Based on the available evidence and the report of a meta-analysis study (33), we cannot deny the protective effects of saffron on memory impairments due to AD or other kinds of disorders. On the other hand, because of the low validity of some clinical trials and several errors or under-reporting in some of them, we cannot also be assured about the saffron protective effects; therefore, we need more clinical studies with a precise design, a large sample size, which include three arms of saffron, an anti-AD drug, and placebo. 

***Depressive disorders***

Based on the estimation of the world health organization (WHO), in the year 2015, approximately 322 million people were suffering from major depressive disorder (MDD) worldwide, and it is still rising now. MDD is more prevalent in females than males of all ages (15 to more than 80 years old) and in all geographic regions (Region of the Americas, African, Eastern Mediterranean, European, South-East Asia, and Western Pacific Region). “Depressive disorders led to a global total of over 50 million Years Lived with Disability (YLD) in 2015. More than 80 % of this non-fatal disease burden occurred in low- and middle-income countries,” WHO reported. This psychiatric disorder can lead to suicide and death, especially in 15-to-29-year-old individuals (34). According to the Diagnostic and Statistical Manual of Mental Disorders, Fifth Edition (DSM-5), these patients usually undergo decreased mood, lose their passions and motivations, or feel worthless. There are also other symptoms that they may experience, such as weight changes (more than 5% of body weight in a month), sleep disorders, etc. Importantly, these problems cause occupational, social, and familial dysfunction. Pharmacotherapy and psychotherapy are routinely used to manage MDD. The other methods are also applied, including ECT, repetitive transcranial magnetic stimulation, vagal nerve stimulation, and more recently, deep brain stimulation (35). 

***Major depressive disorder (MDD)***

Total extract of two parts of *C. sativus* has been assayed against MDD: stigmas and petals. Three clinical studies evaluated crocin, one of the active ingredients of saffron against depression. Moreover, in four studies, saffron was given along with an anti-depressant drug as adjuvant therapy (three of them), or concurrently with another herbal substance, curcumin. All clinical studies on different kinds of depression are randomized and double-blind, except for the cases whose design has particularly been mentioned. The details of treatments in different studies have been summarized in Table 1. 

Two separate trials demonstrated that encapsulated saffron extract had statistically the same effects as imipramine and fluoxetine in patients with mild-to-moderate MDD (diagnosed by DSM-4); furthermore, it exhibited significant effects versus baseline after the first month of treatment (36, 37). Anti-MDD influence of saffron capsules was also compared with placebo, and there was a significant difference between these two groups at the end of the sixth week (38). The side effects of saffron were not statistically different from fluoxetine and placebo, whereas its sedation and dry mouth were significantly lower than imipramine (36, 37). The last three mentioned studies were conducted in the similar time range of January 2002 to February 2004 and in the same place of the outpatient clinic of Roozbeh Psychiatric Hospital (Tehran, Iran), with similar inclusion and exclusion criteria, saffron source, capsule preparation method, and saffron daily dosage (36-38). Given this point, we can deduce from all their results data together: saffron is significantly more effective than placebo in the treatment of mild-to-moderate MDD, comparable to imipramine and fluoxetine, with lower side effects. Pharmacotherapy with synthetic drugs can cause symptom improvement as early as the first 1 to 2 weeks of treatment; however, remission is often observed after 8 to 12 weeks from the treatment initiation (35). Consequently, at least 8-12 weeks are a more reliable treatment duration to assess a new antidepressant compound and compare it with a synthetic drug. In the indicated trials, which have been the first clinical trials in this field, the dosage of the saffron extract was calculated based on an animal study (37); nevertheless, evaluating two or more dosages would have been more informative. Another clinical trial was limited to elderly people who were aged more than 60 and were diagnosed with MDD based on DSM-5. The findings of this study also suggested statistically similar anti-depressant effects between two groups of saffron and sertraline treatment; however, the lack of a placebo group in this trial raises the question of how significant these anti-depressant effects were. Based on the results of this trial, participants in the saffron group experienced no headache, vertigo, and sleep disorders while these side effects appeared in 2-3 of the participants in the sertraline group (39). 

As well as stigmas, the extract of petals of *C. sativus* has been investigated against mild-to-moderate MDD versus placebo and fluoxetine in two different studies. In the first one, the final analyses revealed that Hamilton Depression Scores (HDS) in the test group were significantly lower than the placebo group and also the baseline (40). Furthermore, the extract of petals was as effective as fluoxetine against mild-to-moderate MDD in the second study (41). As the studies suggested, both extracts of stigmas and petals of *C. Sativus* have protective effects on MDD, so these two parts must have one or more active compounds in common. Akhondzadeh Basti and his colleagues showed that the petals and stigmas of this plant had the same effectiveness and also side effects. The effects of both extracts were time-dependently significant in comparison with the baseline (42). A meta-analysis of the randomized trials in 2013 showed that most of these studies have a high quality and based on them, the alleviative influence of crocus extracts on mild-to-moderate MDD was concluded. However, future studies enlisting more subjects, from different countries, and with a longer duration of follow-up were suggested to prove the efficacy of these valuable extracts (43). 

As indicated earlier, there are three clinical studies in which saffron was administered as add-on therapy. Moosavi and his colleagues compared two different doses of saffron extract plus fluoxetine in terms of their effectiveness against mild-to-moderate MDD and side effects. The findings exhibited a significantly better efficacy of saffron with 80 mg/day than 40 mg/day. Side effects, including nausea, dizziness, headache, drowsiness, and insomnia in both groups were statistically the same (44). In the other study, one group was treated with saffron and fluoxetine while the second group was treated with placebo and fluoxetine. Finally, the results represented that there was no significant difference between the two groups neither in the Beck depression scale nor lipid profiles of individuals. One important reason can be the use of 30 mg saffron powder packed in a capsule in this study, despite the other studies which have used 30 mg of the saffron dried hydro-alcoholic extract which may have more potency. A shorter duration of treatment (4 weeks) may also have interfered with the emergence of saffron effects (45). Despite the last study, the addition of a saffron regimen to the previous anti-depressant treatment of patients with persistent mild-to-moderate depression significantly improved their score on clinician-report MADRS (Montgomery-Asberg Depression Rating Scale) in comparison with a placebo group. However, there is also a controversial outcome in the self-report MADRS-S, which showed no significant effects versus placebo (46). To recapitulate these three adjacent therapies, higher doses of saffron (40 and 80 mg/day, based on Moosavi *et al*. study) may be needed for yielding significant effects. 

Crocin is one of the main active ingredients of saffron which is a hydrophilic component and enters into the hydro-alcoholic extract of saffron. Talaei and his colleagues attempted to understand whether crocin is responsible for the anti-depressant effects of saffron in patients suffering from mild to moderate MDD. They designed a randomized and double-blind clinical trial with two arms. Both groups were treated with one of the selective serotonin reuptake inhibitors (SSRIs), whilst they concurrently received either crocin or placebo. Eventually, the results exhibited a significant difference between the two groups, in addition to a significant improvement observed in both groups at the end of the study (47). In the other trial, crocin effects were compared with the total extract of saffron not only on depression but also on health-related quality of life and sexual desire in patients with coronary artery disease (CAD). As it was shown, both crocin and total extract significantly alleviated only depression and quality of life scores, versus placebo and baseline. Crocin was a little effective, but not statistically more effective than the total extract (48). Additionally, another clinical trial on moderate-to-severe MDD cases (unlike other trials) with metabolic syndrome revealed that administration of crocin could significantly mitigate Beck Depression Inventory (BDI) scale, and its ameliorating effects were not accompanied by remarkable lower serum pro-oxidant/anti-oxidant balance in patients (49). 

Some researchers focused on MDD correlated with post-percutaneous coronary intervention (PCI). In this project, the patients with mild-to-moderate MDD who had experienced PCI during the last 6 months of the study were included. The researchers compared the effect of the saffron extract with fluoxetine, as a standard treatment. The results suggested that saffron extract and fluoxetine possessed statistically similar effects. Eighty-five percent of patients in the saffron group and eighty percent of patients in the fluoxetine group reached the complete response (≥50% reduction in the score of Hamilton Depression Rating Scale (HDRS)), although the authors did not separately report the comparative analyses between the final and the baseline of the subjects’ depression score in both groups (50). 

***Postpartum and post-menopausal depression***

The importance of efficient treatment for postpartum depression is more than other kinds of depression because two humans are affected in this case: the mother and her baby; so, any negative consequences would be two-fold more than other depressions (51). As a result, finding an effective and safe medication for breastfeeding mothers would be valuable. With this approach, saffron as a herbal drug was assessed in two randomized and double-blind clinical studies. One of them showed the significant effectiveness of standardized saffron capsules versus placebo and baseline. The findings also demonstrated no significant adverse effects in the saffron group compared with the placebo group. The reported side effects were bleeding gums (one), gastrointestinal disorder (two), hypersomnia (one), insomnia (one), and breast milk reduction (two). There was no side effect in the subjects’ infants as well (52). In addition to this trial, the other study proposed that saffron extract strongly resembled fluoxetine in improving effects on postpartum depression. However, the results were flawed by not reporting the *P*-value between final and baseline scores. There were more cases of headache, dry mouth, daytime drowsiness, constipation, and sweating in the fluoxetine-treated subjects than in the saffron-treated ones, although it was not statistically significant (53). 

There is another study that has been conducted to assay the effects of saffron on postmenopausal women suffering from hot flashes and mild-to-moderate depression simultaneously. The volunteers were followed up by the Hot Flash-Related Daily Interference Scale (HFRDIS) and HDRS at first and every two weeks of study until 6 weeks. The results demonstrated that saffron decreased both scores significantly more than placebo. However, they did not indicate any analysis versus baseline like some prior studies (54). 

Taken together, extracts of stigma and petals of *C. sativus* L. have exerted promising effects on humans against mild-to-moderate depression without any significant side effects. A meta-analysis that has been carried out on seven of these randomized controlled trials (RCTs) in the year 2018 supported this conclusion (55). Findings demonstrated the time-dependent effectiveness of saffron. In one study also dose-dependency of the saffron effects was shown (80 mg/day versus 40 mg/day). Although most of studies used 30 mg/day of extract, evaluating higher dosages in the future studies can be profitable. As the last point, using the hydro-alcoholic extract of stigmas or petals is preferred in order to take advantage of this herb. 

***Comorbid depression and anxiety***

MDD is highly accompanied by other mental disorders, in approximately 75% of cases (56). A recent study demonstrated that anxious distress was diagnosed in two-thirds of MDD outpatients (57). It has also been indicated that this comorbidity can lead to more severity and disability of anxiety in these patients (56). 

The three following randomized double-blind clinical trials have been performed on patients struggling with a mixed anxiety-MDD disorder diagnosed by DSM IV or V. In the first study, although the dried stigmas were used in the saffron group rather than its extract, a much higher dose, about 3-fold more than the usual doses (30 mg/day) was administered. Eventually, a significant difference was found between treatment and placebo in both anxiety and depression scores; however, the statistical analysis of the scores after 12-week treatment in comparison with the baseline has not been reported. The saffron capsules had not been standardized before the study, which reduces the article’s validity. Considering that the performers have chosen a relatively higher dose of saffron stigmas, it would have been valuable, if they had assessed the safety or any adverse reactions (58). In another study, saffron extract had statistically similar effects to citalopram in these patients. Although there was no placebo group, citalopram and saffron had significant effects versus baseline from the second week. The remission rate of the saffron group changed from 6.7% in the second week to 63.3% in the sixth week, while it was from 0 to 86.7% in the citalopram group. It appears saffron had an earlier onset of action. Comparison between side effects of saffron extract and citalopram showed that some adverse effects were less frequent in the saffron group including vertigo, drowsiness, gastritis, anger/rage, and palpitation; however, their differences were not significant (59). Effects of hydro-alcoholic extract of saffron were also evaluated in patients with both type-2 diabetes and mixed anxiety-depression. In this double-blind and randomized trial, subjects were just allowed to take metformin and glibenclamide for managing their diabetes. At the end of the study, the items of anxiety, depression-anxiety, depression, life satisfaction, and sleep disorder were analyzed between saffron extract and placebo groups. Saffron was only effective against anxiety, depression-anxiety, and sleep disorder. Contrary to the above-mentioned studies, saffron extract failed to exert any anti-depressant effects in these cases (60); the reason can likely be the interference of diabetes or its related medications.

The aim of the next clinical study was the evaluation of a standardized saffron extract in young participants (12–16 years old) with a diagnosis of mild-to-moderate anxiety or depression. The cases were monitored by the Revised Child Anxiety and Depression Scale (RCADS) which has several subscales of separation anxiety, social phobia, generalized anxiety, panic, obsessions/compulsions, and depression with two versions of self-report and parent-report. Given this questionnaire, the mean percentage of improvements from the initiation to the end of the trial in the saffron group was significantly more than in the placebo one in the opinion of both patients and their parents; whereas, the percentage of responders to saffron was significantly higher than placebo only from the perspective of the patients themselves. Inconsistency in some results obtained from the parent reports or some mismatches between two reports makes the final decision uncertain (61). Using an expert-based instrument for monitoring could reduce these types of variations and uncertainties.

In the rest of the trials, a diagnosis of anxiety and/or MDD was not necessarily considered an inclusion criterion. Each study has worked on possible anti-anxiety and/or anti-depressant effects of saffron on self-reported mood disorders in a special group of individuals: physically healthy, post-CABG, and diabetic patients. 

Accordingly, the first study investigated the efficacy of two different dosages of the saffron extract on anxiety, mood, and sleep quality in healthy individuals. The volunteers were selected on the condition that they complained about a decreased mood even without any diagnosed mood disorder. This trial revealed that only the higher dosage of saffron extract (28 mg/day) significantly improved the scores of negative effect, depression, and anxiety in comparison with the placebo arm (62). Secondly, investigation into the influence of saffron extract on post-CABG patients under 70 years old demonstrated no significant changes in anxiety-depression and cognition scores after treatment. Surprisingly, despite the declarations in this article and also some other evidence (63-65), the subjects did not show any significant anxiety-depression or cognitive disorder after surgery. It should also be noticed that the number of subjects who completed the study was small (37) (30). In the third study, the possible alleviating impact of saffron on sleep disorder or anxiety of diabetic patients was examined with a very high dose of saffron and a very short duration of treatment. Although there was a significant decrease in the scores of anxiety and sleep disorder in the saffron group compared with the baseline, the demographic data of the participants were not statistically compared between the two groups. The authors reported that 96% of participants were married or 30 % of them had a master’s degree, but their distribution between the two groups was not explained. Moreover, they did not statistically compare the severity of anxiety or sleep disorder of patients at baseline. These factors can interfere with the assessments. Meanwhile, safety evaluations were not reported, considering that saffron was acutely administered in this study (66).

All in all, as a new meta-analysis concluded, saffron could be an effective anti-depressant agent; however, more multi-center and large-scale clinical trials including participants in different ethnic groups are recommended (67). The possible molecular mechanisms of the anti-depressant effects of saffron and crocin have been investigated in some preliminary animal studies, which need a separate comprehensive discussion. For instance, these experiments suggest that an increase in the phosphorylation of cyclic-AMP response element-binding protein (CREB), an elevation of the level of brain-derived neurotrophic factor (BDNF) (68-71), and a reduction in inflammation and oxidative stress (72) might be responsible for these anti-depressant effects.

***Premenstrual syndrome (PMS)***

Some women experience unpleasant symptoms, which can be psychiatric and/or somatic, in the luteal phase of their menstrual cycle. These symptoms disappear as soon as the first days of menstruation. The negative point is that this syndrome disrupts their normal daily activities. It can be prevented by pharmacotherapy with some of anti-depressant drugs or contraceptives (73). However, a survey in the United States showed that 80% of women would prefer non-pharmacological treatments such as supplements (74). So, assessment of the herbal active substances can be valuable. Only one double-blind, randomized and placebo-controlled clinical trial has been conducted to investigate the possible effects of saffron against PMS. Twenty-four women, who received 15 mg of saffron extract twice daily were compared with the twenty-three subjects receiving placebo capsules with the same administration. They were followed up by HDRS and Total Premenstrual Daily Symptoms (TPDS). Although both scores of subjects at the start point were statistically the same, after 2 months, they were significantly lower in the saffron group than the respective ones in the placebo group and also the baseline. As a result, it demonstrated that saffron extract may significantly improve menstrual-related mood disorders as well (75).

***Obsessive-compulsive disorder (OCD)***

The saffron extract was also examined for any positive effects on OCD, an intensively disabling and annoying neuro-psychiatric disorder, which decreases patients’ quality of life. It is usually managed by pharmacotherapy and/or psychotherapy while there are some new methods including electroconvulsive therapy and transcranial magnetic stimulation (76).

Esalatmanesh and her colleagues (2017) studied 46 mild-to-moderate OCD patients based on test revision of DSM-IV and Yale-Brown Obsession Compulsion Scale (Y-BOCS). They were randomly categorized into two groups, one of which received commercial saffron extract (30 mg/day) for 10 weeks. In parallel, the other group received 100 mg/day of fluvoxamine as a control. The final scores were analyzed between two groups after 2, 4, 6, 8, and 10 weeks. There was no difference between the positive effects and adverse effects of these two compounds. Although fluvoxamine is one of the standard treatments for OCD, its effectiveness should have been confirmed at least either by statistical analysis versus its baseline or a placebo group (77). A new similar study has been performed to compare crocin effects (15 mg/day during the first month of treatment, then 30 mg/day for the second month) with fluoxetine (20 mg/day and 40 mg/day, respectively for the first and second month) against mild-to-moderate OCD. Finally, this study also revealed the same findings as the former one with both Y-BOCS and HARS scores with two differences. First, it showed statistically significant effects of both treatments in comparison with their baseline, which makes it more reliable. Second, crocin revealed significantly lower side effects versus fluoxetine (78). 

These two double-blind and randomized studies suggest promising effects of saffron and its constituent, crocin, against OCD; however, using a placebo group and a bigger sample size in both studies would have minimized possible errors and given us a more reliable result. 

***Attention deficit hyperactivity disorder (ADHD)***

This disorder, as its name implies, appears by the inability to concentrate and also overactivity. The onset of this disorder is most often in childhood but if it is not seriously considered, it will remain even until adulthood. The prevalence of ADHD is 5 times more in males than females. It has been reported that behavioral interventions at least 8 weeks before main pharmacotherapy can lead to a better response to the treatment (79). ADHD has been regarded as another target for saffron effects. It possessed comparable effects to methylphenidate in 6–17 year-old patients. Both saffron and methylphenidate capsules were gradually administered to the maximum dose of 20 mg/day for children lower than 30 kg and 30 mg/day for children higher than 30 mg/day. The participants were monitored by two versions of the teacher- and parent-reported ADHD Rating Scale (ADHD-RS). The duration of this double-blind and randomized clinical trial was 6 weeks with a sample size of 50. Treatments with saffron and methylphenidate caused respectively 52% and 56% reduction in the teacher-reported version of ADHD-RS. In this trial, a placebo arm was not considered. The experienced side effects of saffron were less than methylphenidate, but its difference was not statistically significant. It is too soon to be assured about the efficacy of saffron extract against ADHD (80). Accordingly, more long-term placebo-controlled trials are suggested in this field. 

***Sleep disorder***

Four randomized, double-blind, and placebo-controlled clinical trials have recently been published, which applied saffron extract against insomnia. Lopresti and his colleagues studied 63 healthy subjects with self-reported sleep insufficiency in two categories including those treated with either saffron (14 mg, BID) or placebo for 28 days. They observed significant time-dependent enhancement in the scores of the Insomnia Severity Index (ISI), Restorative Sleep Questionnaire (RSQ), and some domains of the Pittsburgh Sleep Diary (PSD) such as sleep quality and the number of awakenings after sleep onset. They additionally demonstrated that these positive effects may not associate with mood improvement because the scores on depression, anxiety, and stress scale-21 failed to change significantly after treatment of subjects with saffron when compared with the placebo group (81). In an updated version of this study, more subjects (120) and two different saffron doses (14 and 28 mg, one hour before sleep) were assessed. Similar to the previous study, positive effects of saffron against insomnia were observed only in some aspects of sleep disorder, and two doses of saffron did not show significantly different efficacy. Besides, saffron administration elevated salivary evening melatonin levels while exerting no remarkable effect on salivary evening cortisol (82). In the third study, a lower dose of saffron (15.5 mg/day) within a longer duration (6 weeks) was considered. In this trial, similar findings were obtained although the participants suffered from mild-to-moderate anxiety, in addition to a mild-to-moderate primary sleep disorder. The results represented a significant positive change only in some domains of the Leeds Sleep Evaluation Questionnaire (LSEQ) and Pittsburgh Sleep Quality Index (PSQI) including ease of getting to sleep, sleep quality, latency, duration, and PSQI global score. Furthermore, the authors assessed the subjects’ quality of life by a short form 36 items (SF-36) questionnaire, no domains of which were affected by saffron treatment (83). Additionally, intranasal administration of an oil-based formulation containing saffron concomitant with lettuce seeds and sweet violet (in the same ratio), in the other clinical trial, resulted in similar effects. After 4 and 8 weeks of treatment (2 drops every noon and evening), participants had significantly lower ISI scores and remarkable improvement only in the domains of sleep quality, duration, and global scores of PSQI (84). In all of these studies, placebo groups depicted significant amelioration in some aspects of the participants’ sleep in comparison with their baseline. This might suggest a notable psychological impact on insomnia treatment.

Overall, these present trials have displayed significant improvements in sleep disorders in humans; however, studies with larger sample size, probably higher doses of saffron, and a longer duration of treatment will provide us with a larger body of evidence to consider it as a hypnotic substance. 

***Pain***

Saffron was clinically tested against two different kinds of pain, dysmenorrhea and late-onset muscle soreness after exercise. Both studies were double-blind, randomized, and placebo-controlled. Although these two types of pain are different, they have a common part in their pathophysiology, which is a rise in prostaglandins, thereby responding to non-steroidal anti-inflammatory drugs (NSAIDs) (85, 86).

In the trial assessing saffron effects against muscle soreness, three groups of subjects were considered: saffron (300 mg/day of dried saffron stigmas), indomethacin (75 mg/day, TID), and placebo (lactose capsules). These capsules were administered 7 days before and 3 days after the eccentric exercise. This kind of soreness is usually concomitant with muscle stiffness, decreased muscle strength, increased creatine kinase (CK), and lactate dehydrogenase (LDH) in serum. Findings demonstrated that the maximum isometric and isotonic force of individuals in the saffron and indomethacin group were significantly higher than placebo 24–72 hr after exercise. In addition, the plasma level of CK and LDH was significantly lower in these two groups versus placebo after 24 hr in the saffron group and after 48 hr from the exercise in the indomethacin group. The level of pain in subjects was also evaluated by Talag Scale Perceived Pain, which showed the same changes as CK and LDH levels (85).

The authors of another study used saffron against primary dysmenorrhea in combination with two other herbal compounds. Each test capsule contained 500 mg of highly purified saffron, celery seed, and anise extracts (SCA). One hundred eighty volunteers were categorized into three groups of the test, mefenamic acid (250 mg) and placebo. All participants took one related capsule every 8 hr for 3 days from the onset of menstruation or pain, for 3 cycles. The severity and duration of pain in the subjects were monitored via a visual analog pain intensity scale at months 2 and 3. The participants who received SCA experienced significantly lower pain than the placebo groups. They were also allowed to take additional pills if they had intolerable pain; however, the authors avoided including the scores of these patients in data analysis because of possible interference with the main regimens. The results of this part showed that subjects treated with the placebo intended to take significantly extra pills than the two other groups. It implies that this group had significantly more intensive and intolerable pain in comparison with SCA and mefenamic acid groups (86). 

In an animal model, the efficacy of safranal and crocin, two main active ingredients of saffron extract has been revealed on inflammatory pain (87). Saffron likely has alleviative effects on these pains by reducing prostaglandin release. 

***Cerebral ischemic stroke***

Recently, the protective effects of the saffron extract have been investigated on 39 patients with an acute cerebral ischemic stroke. While they were receiving the stroke-specific care, a random half of them were given 100 mg of saffron extract, twice daily, for four days in the hospital, and then 50 mg four times a day for three months. Short-term and long-term assessments represented that saffron significantly lessened the stroke severity and the functional impairment induced by this event in comparison with the control group. It also diminished serum levels of neuron-specific enolase (NSE) and S100 noticeably. BDNF level was significantly elevated in the blood after saffron treatment. These effects show that saffron can ameliorate the transient neurological damages of cerebral stroke. This trial was neither placebo-controlled nor double-blind (88). Based on some animal studies, the anti-ischemic effects of saffron ingredients could be due to its antioxidant activities (22, 89). 

***Multiple sclerosis (MS)***

There are too limited clinical data about the effects of saffron and its constituents against this autoimmune disease. Only one clinical study has revealed the anti-oxidative and anti-inflammatory effects of crocin in patients suffering from MS. In this trial, half of the subjects received crocin (15 mg, BID) and the other half were treated with a placebo. Although the results demonstrated that crocin markedly reversed serum levels of malondialdehyde (MDA), as a lipid peroxidation indicator, total antioxidant capacity (TAC), IL-17, TNF-α, and DNA damage in comparison with the placebo-treated patients, no factor related to MS severity was assayed to show how effective crocin could be on the subjects’ symptoms or their disease severity (90). This trial may encourage scientists to clarify the possible correlation of these protective effects with the clinical MS severity and the quality of life in these patients. 

***Metabolic disorders***

All clinical trials in this section including metabolic syndrome, diabetes, and obesity sub-topics are randomized, double-blind, and placebo-controlled except for one single-blind and two triple-blind studies.

***Metabolic syndrome (MetS)***

When signs of the three disorders including hyperglycemia, hypertension, and hyperlipidemia are diagnosed all together in a patient, it is recognized as metabolic syndrome. This disorder is highly prevalent around the world and increases the risk of cardiovascular diseases and type-2 diabetes. Animal models have represented the saffron protective effects against different components of MetS. It possessed hypolipidemic, anti-obesity, anti-hypertensive, and hypoglycemic effects through different mechanisms (91, 92). There are also human trials related to this part. 

Since this syndrome is a risk factor for cardiovascular diseases (CVD), saffron effects were evaluated on some special related heat shock proteins (HSPs) in the human trials. HSPs increase in stressful conditions, such as high blood pressure and hyperlipidemia and it has been revealed that there is a close correlation between HSPs and CVD. That is, they may have a role in plaque formation and atherosclerosis (93). Two clinical trials were conducted to evaluate whether saffron can diminish metabolic syndrome-induced higher levels of HSPs. Trials were both double-blind and placebo-control; however, one of them worked on dried saffron (50 mg, BID) and the other one assessed crocin (15 mg, BID). The study on saffron had a longer duration (12 weeks vs 8 weeks) and more subjects (105 vs 60). The results revealed that saffron significantly decreased HSP 27 and 70 (not HSP 60 and 65) after 12 weeks, whereas placebo and non-placebo controls did not significantly change. The other study showed crocin failed to reduce HSP 27 and hs-CRP after 8 weeks (94, 95). The justification for this paradox can be the shorter duration of treatment, fewer subjects in the study on crocin, or its lower dosage. It is also possible that crocin is not responsible for saffron’s effects against HSPs elevation developed by metabolic syndrome. Nevertheless, this hypothesis needs more investigations to be confirmed. 

Another study that worked on possible crocin effects on the oxidation/anti-oxidation balance in patients with MetS represented that crocin significantly reduced this balance after 8 weeks, while no remarkable change was observed in metabolic factors including lipid profile and blood glucose (96). It is concluded that the oxidative process does not probably play an essential role in the pathophysiology of the MetS to be improved by the anti-oxidative effects of saffron. Two other studies also demonstrated the controversial effects of dried saffron stigmas and its active ingredient, crocin, against serum leptin level and cholesteryl ester transfer protein (CETP), respectively. Zilaee and her colleagues compared saffron effects (100 mg/day for 12 weeks) on lipid profile and serum leptin with placebo in 76 participants suffering from MetS. Leptin is a hormone secreted from adipose tissue and its elevated level can lead to appetite control and weight gain prevention. The intervention with saffron caused an increased level of this hormone to the significance borderline in comparison with the baseline. The difference between changes in leptin levels in the test and placebo groups was also significant. However, the lipid profile of patients was not significantly affected by saffron except for low-density lipoprotein- cholesterol (LDL-C) level, which was remarkably reduced after 12-week treatments with saffron (97). In the study of Javandoost and colleagues, although administration of 30 mg/day of crocin diminished CETP levels in the patients with MetS versus baseline, failed to alter the CETP and lipid profile compared with the placebo group after 8 weeks. The serum level of CETP depends on fat mass and it decreases high-density lipoprotein-cholesterol (HDL-C) (98). Furthermore, there is another clinical research assessing saffron effects on serum levels of cytokines and MetS-related items in forty-four patients. To reach this goal, 100 mg/day of saffron was given to half of the subjects and the rest of them received identical placebo capsules. After 12 weeks, the level of interleukin-1α (IL-1α), IL-1β, IL-2, IL-4, IL-6, IL-8, IL-10, tumor necrosis factor-alpha (TNF-α), monocyte chemoattractant protein-1 (MCP-1), interferon-gamma (IFN-ɣ), endothelial growth factor (EGF), and vascular endothelial growth factor (VEGF) was measured as well as lipid profile, blood glucose, and blood pressure. Despite the fact that only IL-6 and VEGF levels increased in comparison with the baseline by saffron, none of the other cytokines was significantly changed when compared with the placebo. Besides, among MetS items only total cholesterol and LDL remarkably decreased versus the baseline and the placebo (99). More sample size would result in a significant change in some cytokines, so now we cannot infer the correlation between IL-6/VEGF and LDL/cholesterol alterations.

Metabolic syndrome can be developed by some antipsychotic medicines. Some researchers designed a triple-blind clinical trial to understand whether saffron extract and crocin prevent olanzapine-developed MetS or not. They enrolled 66 patients with schizophrenia who met the criteria of receiving 5-20 mg/day of olanzapine and then randomized them into three groups of saffron extract (30 mg/day), crocin (30 mg/day), and placebo. Finally, data analysis exhibited that 27.3% of patients in the placebo group developed MetS while this percentage was 9.1% and 0%, respectively in crocin and saffron extract groups. The average amounts of any parameters including blood pressure, lipid profile, and insulin resistance did not change significantly after 12 weeks, except for fasting blood sugar, which decreased by crocin and total extract of saffron (100). 

Taken together, the studies have obtained controversial results about the effectiveness of saffron against metabolic syndrome. The studies with longer duration or more subjects caused more positive effects on these patients. It illustrates that saffron likely needs more time or even higher doses to be effective against metabolic syndrome. Thus, these points should be considered in future studies. 

***Diabetes***

Diabetes is one of the most prevalent diseases, which has globally led to a high rate of deaths, as reported in the year 2015, five million people lost their lives due to diabetes. Moreover, two major groups of complications may appear in these patients: micro-vascular (such as ocular, renal, neural, etc.) with the prevalence of 50% of whole patients, and macro-vascular complications, which have been reported in 27% of them (101). Up to now, clinical trials are investigating the possible effects of saffron against both metabolic parameters of diabetes and some of its complications. But in this part, we will report only the topics related to the metabolic aspects of diabetes, and the other topics about the complications will be discussed in their related parts.

One of the most recent clinical trials in this field applied saffron extract to evaluate whether it will mitigate metabolic factors in diabetic patients after 3 months. They found that fasting blood sugar (FBS), cholesterol (Chol), LDL, LDL/HDL ratio, and alkaline phosphatase (ALP) considerably declined in comparison with the placebo group by intake of 30 mg/day of saffron extract. However, insulin level, insulin resistance scores (HOMA-IR), renal and hepatic factors as well as Glycated hemoglobin A1c (HbA1c), HDL, and triglyceride (TG) did not have any significant changes (102). Consequently, the 3-month effect of the saffron extract on blood glucose in diabetic patients is not possible through increasing insulin secretion or decreasing insulin resistance, which is a crucial factor in the pathophysiology of diabetes. Although the insulin level failed to change in the previous study by 30 mg/kg of saffron, its administration at 100 mg/day could significantly slim down the diabetic patients’ serum insulin level and HOMA-IR (not yet HbA1c) after 8 weeks, in addition to improving their lipid profile and decreasing the serum level of hepatic enzymes. Simultaneously, the sleep quality of these subjects, their quality of life, and the Beck depression scale were enhanced after this intervention (103). The treatment duration in two other studies was the same as the last-mentioned trial (8 weeks). In one of them, which was a triple-blind trial, saffron extract (30 mg/day) did not noticeably change any factors (lipid profile and blood glucose factors) except FBS, based on the time×intervention effect (104). The other clinical study, which was a single-blind trial also demonstrated no remarkable effectiveness of saffron (1 g in combination with 3 glasses of black tea) on FBS, HbA1c, and insulin; in contrast, lipid profile was significantly alleviated (105). Although in this study placebo group also received 3 glasses of black tea, it is not clear if black tea had an interfering effect with saffron (perhaps with its absorption or effectiveness). Therefore, it seems that the intervention group is not completely comparable with the control group, and saffron should have been assessed separately.

The effect of saffron was also investigated on pre-diabetic overweight or obese participants in the dosage of 15 mg/day for 8 weeks. After the treatment period, the analyses exhibited that saffron could significantly decrease FBS and HbA1c, while it was not effective against lipid profile (106). It should be noticed that the FBS levels of these volunteers were not as high as diabetic patients. As a result, saffron effectiveness with such a low dose in these individuals was not impossible.

***Obesity***

Several animal studies have shown the anti-obesity effects of saffron in different ways, which is reviewed by Mashmoul and her colleagues (2013). In this review, the authors listed four main mechanisms of saffron: anti-oxidative and anti-inflammatory effects in adipose tissue, appetite reduction, decreasing fat absorption through pancreatic lipase inhibition, and hypolipidemic/hypoglycemic effects (107). There are fewer clinical trials on this part. 

Anti-obesity effects of saffron were assayed in healthy women (25-45 years old) by a randomized and double-blind study. Sixty mildly-overweight women (25 kg/m^2^ <BMI< 28 kg/m^2^) were enrolled in this trial, half of which received 88.25 mg of saffron extract twice daily, while the other half received similar placebo capsules. After this 8-week treatment, volunteers lost approximately 1 kg of their weight. This change was significant in comparison with the placebo group whereas it was not statistically meaningful against the baseline. Moreover, the frequency of snacking in the subjects was significantly reduced compared with the placebo. The body composition of the volunteers was also analyzed and the results revealed that only thigh circumference markedly decreased. The limitation of this trial is that the stress/anxiety level, daily calorie intake, and daily activity of each subject, which are very important factors in weight change, were not assessed (108). Another double-blind clinical trial was performed on 75 CAD patients between the ages of 40 and 65 to evaluate the effects of saffron and crocin on patients’ weight and body composition. Three arms of saffron extract (30 mg/day), crocin (30 mg/day), and placebo were randomly designed. The final analysis after 8 weeks demonstrated a significant reduction in weight, fat mass, and increment of satiety and fullness feeling by saffron total extract and crocin; however, total extract possessed more potent influences than crocin (109). Although the dosage of saffron extract in this study was much lower than the previously mentioned study (30 mg versus 176.5 mg per day), its effects on weight in this study were more (about 2.31 kg versus 1 kg). The sex, age average, and health condition of subjects are mainly different between these two studies and it might be the origin of this paradox. Furthermore, the lipid profile and FBS levels of participants were also measured in the second study, where none of the items were affected significantly by saffron extract or crocin. Another clinical trial, which was also randomized, double-blind, and placebo-controlled, depicted some controversial results. That is, the intake of 30 mg/day of saffron for 12 weeks failed to exert any anti-obesity effects in the depressed overweight women (BMI ≥ 25), and it was only effective against the depression score of subjects solely after 12 weeks (110). This study and the first one both were conducted on adult overweight women. Despite the longer duration of the last study, its saffron dosage was about 6-fold lower than the first one, which may be the reason for its negative final results. Overall, the anti-obesity and appetite-reducing effects of saffron need to be confirmed by larger clinical studies and different doses of saffron.

***Fatty liver disease***

Recently, a double-blind clinical trial has been conducted to evaluate a relatively high dose of saffron (100 mg/day) in cases diagnosed with non-alcoholic fatty liver disease (grades 1-3). In this study, 76 volunteers were randomly categorized into two groups: saffron- and placebo-treated. After a 12-week intervention, the findings demonstrated that the level of some factors representing inflammation and oxidation including hs-CRP and MDA were significantly reduced, and TAC was elevated in the saffron-treated sample blood versus the placebo group. In contrast, hepatic enzymes, TNF-α, and adiponectin levels were not considerably affected. The authors assessed leptin level as an indicator of non-alcoholic steatohepatitis severity, which was remarkably diminished by saffron treatment (111). However, longer trials with a higher scale should be performed to investigate the question of whether saffron can lower the grade of this disease or at least slow down its progress; otherwise, these data not only are controversial but too limited to convince scientists of clinically applying saffron in these cases. 

On the whole, three meta-analysis studies came to different conclusions about the effects of saffron treatment on blood glucose and lipid profile of participants (112-114), which display its controversial effects or at least insufficient data on this subject. 

***Ocular diseases***

Among various ocular diseases, age-related macular degeneration is known as an important cause of irreversible loss of sight. This disease is associated with both genetic and environmental factors. Now, anti-vascular endothelial growth factor (anti-VEGF) medicines are applied to decelerate the disease process, although they cannot be completely managed yet. Saffron has been considered for its positive effects against this ocular disease and also some other ones including primary open-angle glaucoma and diabetic maculopathy. It is suggested that its possible mechanisms are related to its anti-oxidative, anti-apoptotic, anti-inflammatory, neuroprotective, anti-hypertensive, and anti-atherogenic effects (115).

***Age-related macular degeneration (AMD)***

The first clinical study in this field was published in 2010 and assessed the effects of saffron on retinal function in patients with bilateral early-AMD via focal electroretinogram (f-ERG). Then, two similar works were carried out again on these patients in 2012 and 2013. In the first study, which was a cross-over clinical trial (double 3 months), a saffron group was compared with a placebo group and its baseline, the second one was open-label, with a longer treatment duration (15 months) and without a placebo group. In the third one, only two groups of patients with different genotypes (complement factor H (CFH) and age-related maculopathy susceptibility 2 (ARMS2)) were compared every 3 months for 12 months to determine whether saffron effects are genotype-dependent or not. Findings of all the trials demonstrated that daily administration of 20 mg of saffron resulted in a significant improvement in the retinal function of patients after 3 months and its effectiveness was not related to the genotype of patients and was stable for at least 15 months. These three clinical trials were a kind of preliminary ones because they had a small sample size (in order of years: 25, 29, and 33) (116-118). The next clinical trial was conducted in a slightly different way and it accordingly revealed some different results. It was performed on forty wet and dry AMD patients and controlled with a placebo. The wet AMD patients were monthly injected with intravitreous bevacizumab concurrently. After 6 months, macular thickness significantly decreased only in wet AMD patients, which was probably because of potentiation in bevacizumab effects, and ERG amplitude was remarkably ameliorated in both types of AMD after 3 months in comparison with the placebo; however, this change surprisingly did not last 6 months. This result is contrary to the findings of prior studies, while the higher dose of saffron (30 mg/day) was used. It may be due to the different types of AMD in the subjects or the different study designs (a double-blind trial) from the aforementioned studies (119). Furthermore, Riazi and colleagues (2016) also investigated the possible protective effects of saffron on patients suffering from dry AMD. They administered 50 mg/day of saffron stigmas to twenty-nine patients, and twenty-five subjects received placebo capsules, for 3 months. Finally, the degree of mean corrected vision of subjects was significantly reduced and their mean contrast sensitivity was noticeably enhanced in comparison with the placebo and the baseline. This study has applied the highest dose of saffron among all similar studies (120). The last trial which was a randomized, double-blind, placebo-controlled, and cross-over study assigned 100 participants with mild-to-moderate AMD, and they were allowed to continue taking any kind of eye-related supplements concurrently with the main trial treatment. Seventy percent of participants were receiving Age-Related Eye Diseases Study (AREDS) supplements. Saffron was administered with the dose of 20 mg/day for 3 months twice. During the first 3 months, saffron was given to the first half of the subjects and during the second 3 months, it was given to the other half of the participants. Evaluation of best-corrected visual acuity (BCVA) and multifocal electroretinogram (mfERG) in the subjects represented that saffron can modestly preserve retinal function in AMD patients compared with the placebo (121); however, using other supplements along with saffron leads to uncertainty about the effects of saffron; in other words, it is not clear if the observed protective effects are the consequence of a potentiation, an additive effect or a synergism between the effects of saffron and other supplements. 

Another clinical research was conducted to assess the possible effects of *Crocus sativus* on gene-related macular dystrophy, not an age-related one. Thus, it was discussed separately in this part. Patients with Stargardt macular dystrophy (SMD) are suitable candidates for anti-oxidative therapy because they undergo retinal oxidative injuries due to a mutation in the ABCA1 gene. As a result, Piccardi and his colleagues evaluated the effects of daily administration of saffron (20 mg/kg for 6 months) on these patients in a two-period cross-over, double-blind, and randomized trial. Finally, different ophthalmic assessments such as fERG and visual acuity measurements indicated the potential effectiveness of saffron in preventing disease progression, and the authors believe that it could be considered for longer studies (122). Considering that there are nowadays novel therapeutic approaches to the treatment of gene-related diseases, herbal supplementation may be mainly helpful in decelerating disease complications.

***Primary open-angle glaucoma (POAG)***

The effect of the aqueous saffron extract was investigated against stable primary open-angle glaucoma as a complementary treatment to timolol and dorzolamide in a randomized pilot study. Two groups of test (30 mg/day of saffron extract for one month) and placebo were compared in terms of intraocular pressure (IOP) every week and after a one-month wash-out period. Based on the results, aqueous extract of saffron significantly reduced the IOP of patients after the 3rd and 4^th^ weeks, and it returned to the baseline level after the wash-out period. It shows that saffron extract can boost the main treatment of glaucoma. This trial was performed with a total of 34 subjects and its findings need to be confirmed by larger studies (123).

***Diabetic maculopathy (DM)***

This pathologic condition is a consequence of high blood glucose and vascular damage in the retina. A double-blind, randomized, and placebo-controlled trial evaluated the possible effects of crocin on this complication in 60 patients. Two doses of crocin were administered for 3 months and compared with a placebo group: 5 mg/day and 15 mg/day, which were lower than its most usual clinical doses. The performers measured not only ocular parameters but also FBS and HbA1c and some other renal, hepatic, and elemental factors in serum. All participants were receiving bevacizumab and most of them were on anti-diabetic medication while receiving crocin. Final results exhibited that the higher dose of crocin significantly diminished central macular thickness, the logarithm of the minimum angle of resolution, FBS, and HbA1c, whilst these changes with the lower dose of crocin were not statistically significant except for FBS. The other measured factors did not significantly alter (124). This study showed promising effects of crocin against diabetic maculopathy as adjuvant therapy for bevacizumab and anti-diabetic drugs. 

***Urogenital disorders***

*Sexual dysfunction*

In a study, Safarinejad and colleagues compared the possible effects of 60 mg/day of dried petal extract of saffron with the effects of 50-100 mg sildenafil in 307 men with erectile dysfunction (ED). They designed an open-label, randomized, and cross-over study. The patients in the sildenafil group took the pill (50 mg) when needed one hour before their sexual intercourse, and increased it to 100 mg if the lower dose was not effective. After treatments, the subjects were assessed with four questionnaires related to ED. The final analyses comparing the saffron group with both baseline and sildenafil groups showed that saffron failed to exert any significant effects against different aspects of erectile dysfunction (125). Although the sample size of this study was large, being open-label and not considering a placebo group are two its weak points . It is noteworthy that in a pilot study, 200 mg/day of saffron, about 3.3-fold higher dose than the prior study, within only 10 days could significantly enhance the rigidity and tumescence of the penis and the total scores of the International Index Of Erectile Function Questionnaire (IIEF-15) in 20 men suffering from ED (126). Furthermore, another study which was a randomized, double-blind, and placebo-controlled clinical trial tested a topical gel formulation of saffron (1%) for one month, against diabetes-induced ED, with a sample size of 50 individuals. Before and after treatments, subjects were evaluated by IIEF-15. This saffron gel represented a significant increment in IIEF-15 scores (127). In addition to possible errors in any of these studies, which could be the reason for controversies between the last two studies and the first one, the possible lower bioavailability of saffron in the first study might be the reason for saffron’s ineffectiveness. In the first study, oral saffron capsules (60 mg/day) were administered to the patients, while in the second and third trials, respectively a much higher dose of oral saffron (200 mg/day) and a topical saffron gel were used. 

Sexual dysfunction is also one of the adverse effects of some medications like SSRIs and it can decrease the patients’ adherence to their drug therapy. Two randomized, double-blind, and placebo-controlled trials were designed to examine if stigma or petal extract of *C. sativus* can improve fluoxetine-induced sexual dysfunction. They enrolled 36 men (128) and 38 women (129) who had become stable on 40 mg/day of fluoxetine at least 6 weeks before the study and suffered from sexual dysfunction. The authors found out that extracts (30 mg/day for 4 weeks) were significantly effective against arousal, lubrication, and pain scales of Female Sexual Function Index (FSFI) (129), and erectile function and intercourse satisfaction scales of IIEF in men (128). However, orgasmic function, overall satisfaction, and sexual desire neither in the men nor in the women were noticeably affected (128, 129). 

***Infertility***

Two clinical trials have tested the possible effects of saffron on semen parameters in two types of reproductive disorders leading to infertility: idiopathic oligoasthenoteratozoospermia (OAT) and varicocele. None of these studies demonstrated the positive effects of saffron on infertile patients (130, 131). Both trials were placebo-controlled and randomized, but the study of Safarinejad and colleagues was also double-blind and conducted with 260 patients suffering from OAT. In this study, saffron was administered at the dose of 60 mg/day for 26 weeks (130). In the other study, 60 mg of saffron powder was given every other day to half of assigned patients 3 days after varicocelectomy and for 24 weeks. Only the sperm motility was improved by saffron and the rest of the parameters including volume, morphology, and count of sperms did not noticeably alter after treatment (131). 

***Cardiovascular disease***

*Atherosclerosis*

Some researchers investigated whether saffron can reduce plasma microRNA-21 in atherosclerotic patients with 30% to 70% occlusion in the coronary vessels. MicroRNA-21 is a regulating factor for gene expression in the endothelial cells and rises in some pathological situations such as cardiovascular diseases, thus it can be an indicator for atherosclerosis and endothelial dysfunction. This randomized, double-blind, placebo-controlled clinical trial was designed based on this purpose. The participants were on their usual treatment, but about half of 63 patients were administered 100 mg/day of saffron for 6 weeks and the other half received placebo capsules. They finally demonstrated that saffron significantly reduced the microRNA-21 level without any significant change in lipid profile, FBS, and blood pressure (132). Consistently, the second study, which was an 8-week, randomized and placebo-controlled trial, indicated that 30 mg/day of aqueous extract of saffron failed to affect the measured factors in CAD patients (including the serum level of ox-LDL and the expression level of Sirtuin-1 (SIRT-1), 5’-adenosine monophosphate-activated protein kinase (AMPK), Lectin-like oxidized LDL receptor 1 (LOX-1), nuclear factor kappa-light-chain-enhancer of activated B cells (NF-κB), and MCP-1) except the serum level of MCP-1. These factors are all involved in the pathophysiology of atherogenesis. In contrast, in a similar study, the third arm, which received 30 mg/day of crocin, showed significant improvements in mentioned factors (133). These conflicting results might rise from any differences (such as potency) between saffron total extract and its active ingredient, crocin, because another active ingredient of saffron, crocetin, also exerted positive effects in the other study on subjects with CAD. In this double-blind clinical trial, patients were randomly administered either crocetin (10 mg/day) or placebo capsules for two months. Based on the findings, not only was the expression level of the aforementioned factors alleviated, but also it could remarkably decrease LDL/HDL level, serum circulating homocysteine, adhesive molecules such as intercellular Adhesion Molecule 1 (ICAM-1) and vascular cell adhesion protein 1 (VCAM-1), and heart-type fatty acid-binding protein (hFABP), as a heart-specific biomarker of ischemia. Furthermore, crocetin could even significantly reduce systolic and diastolic blood pressure and BMI in the patients versus the placebo group (134). It seems that the active ingredients of saffron have been more effective than its total form; nevertheless, more data need to be obtained for final comprehensive analyses. 

***Hypertension***

Several *in vivo* studies have shown the anti-hypertensive effects of saffron and its active ingredients such as crocin and safranal (135-138). “Anti-hypertensive” is a better word for this effect than “hypotensive” because it has been shown that saffron is a blood pressure modulator, which means it will not reduce normal blood pressure (139). In a double-blind and placebo-controlled trial, hemodynamic parameters were measured in 30 healthy volunteers. They received either 200 mg or 400 mg saffron or placebo tablets in three different groups for 7 days. After this short-term treatment, only standing systolic and standing mean blood pressure were significantly lower than their baseline, not in the sitting state, and not in comparison with the placebo group (140). Another clinical trial (randomized, single-blind, and placebo-controlled) has assessed the possible effects of saffron on ICAM-1 level and blood pressure in diabetic patients. These subjects were enrolled with no specific regard to their blood pressure status. The final results showed that saffron supplementation did not have a significant effect on either of the two measured factors versus placebo; however, the average systolic blood pressure after an 8-week treatment with saffron was meaningfully lower than the baseline. In this trial, forty-four diabetic patients were trained to traditionally stew one gram of saffron stigmas in black tea for 10 min and then drink it (141). It seems that this method of administration was not under strict control and it may have caused a high variation from one person to the other. Moreover, the patients consumed the aqueous extract of saffron, but it was not determined how much of the active ingredients finally was taken by each of them or whether the active ingredients of saffron were completely stable in this situation (especially due to using heat). 

In contrast to the above-mentioned trials, the study of Mahmoudi *et al*. was conducted on 60-70 year-old men suffering from high blood pressure. Their study demonstrated that daily intake of 200 mg saffron for 12 weeks could significantly lower the systolic and diastolic blood pressure of the participants in comparison with both their baseline and a control group. They additionally revealed that this effect may be associated with a decrease in the peripheral vascular resistance via elevation in serum atrial natriuretic peptide, nitric oxide, Adiponectin, and, reversely, a reduction in serum Endothelin-1 (142).

Overall, although more and larger clinical trials are recommended for concluding the possible effects of saffron against hypertension, the present studies showed promising effects of saffron on blood pressure specifically in hypertensive patients rather than normotensive ones. 

***Gastrointestinal disease***

There are various animal and cell culture studies about the effects of saffron and its constituents on gastrointestinal disorders such as peptic ulcers, gastrointestinal cancers, and colitis; however, far fewer clinical trials have been conducted in this field (143). 

***Irritable bowel syndrome (IBS)***

IBS is a psychosomatic disorder that was considered a target for possible protective effects of saffron. Akhondzadeh and his colleagues performed a double-blind randomized clinical trial. They compared 30 mg/day of saffron with 40 mg/day of fluoxetine in two groups of thirty-five volunteers with diagnosis of IBS. From the second week of this 6-week treatment, saffron could significantly enhance the quality of life of patients and also decreased their comorbid depression and anxiety scores after 4 weeks (144). Although this investigation suggested promising effects of saffron against IBS, not including a placebo group reduced the strength of this trial. Therefore, larger placebo-controlled clinical trials are necessary. 

***Ulcerative colitis***

This type of inflammatory bowel disease can be accompanied by oxidative stress, which can accelerate disease progression. Saffron, as an anti-oxidant substance, was applied against ulcerative colitis in a randomized, double-blind, and placebo-controlled clinical trial. The findings implied this compound in a relatively high dose (100 mg/day) could weaken the severity of the disease (based on a simple clinical colitis activity index questionnaire), augment TAC and enzymatic anti-oxidation through the increment of Glutathione Peroxidase (GPX), and Superoxide Dismutase (SOD) level. However, the serum level of MDA did not significantly change in the saffron group in comparison with the placebo (145). Although this clinical trial has revealed promising effects against ulcerative colitis, these data do not suffice for coming to any certain conclusion about the practical application of saffron in this case. 

***Respiratory disease***

Among different respiratory disorders, the impact of saffron was clinically assayed on mild-to-moderate allergic asthma. Because of the inflammatory nature of this disease and the anti-inflammatory effects of saffron, it was predicted to be effective against allergic asthma. Two clinical trial articles were published in this field, which had the same treatment protocol (100 mg/day of saffron for 8 weeks) and sample size (80 individuals). Both were placebo-controlled and randomized (146, 147). The study of Zilaee and her colleagues, which was double-blind, demonstrated that saffron significantly improves some factors such as the frequency of nocturnal breath shortness, asthma causing an inability to sleep, activity limitation, and use of salbutamol spray in comparison with the placebo and the baseline. The eosinophil level of saffron-treated subjects was significantly lower than the baseline (147). In another study, which was triple-blind, the level of hs-CRP, anti-HSP70, and spirometry parameters were measured. hs-CRP and anti-HSP70 were significantly decreased and forced expiratory volume in the first second (FEV1), forced vital capacity (FVC), FEV1/FVC ratio, and forced expiratory flow 25-75% (FEF 25-75) in the saffron group were remarkably higher than the placebo group (146). These findings suggest the ameliorative effects of saffron on allergic asthma, although it needs more studies to confirm these findings.

***Musculoskeletal disorder***

Up to now, in this category, only two relevant clinical trials (randomized, double-blind, and placebo-controlled) have been conducted, one of which investigated saffron effects against rheumatoid arthritis (148), and the other one assayed crocin effects on osteoarthritis (149). Both of these diseases are chronic and inflammatory; as a result, saffron and its ingredients, as potential anti-inflammatory agents, could be promising candidates to be considered for lowering the severity and progression of these diseases. 

As the first study depicted, the subjects who received 100 mg/day of saffron experienced significantly less pain intensity after 12 weeks based on a visual analog scale (VAS) in comparison with the placebo group. Moreover, the number of tender and swollen joints, the arthritis activity score, and the serum level of ESR were remarkably decreased in these patients. On the contrary, some other related inflammatory and oxidative parameters including TNF-α, INF-ɣ, hs-CRP, MDA, and TCA remained unchanged when statistically compared with placebo (148). However, the second study on osteoarthritis demonstrated that 15 mg/day of crocin could significantly diminish CRP level after 4 months while they did not observe any significant difference in pain severity (by VAS) between the two groups. This trial also revealed that crocin could regulate the immune system and shift T-helper cells (Th) from Th-17 to regulatory Th (149). The contradictions between the results of these two trials can originate from their basic disparities in study design including the main targeted disease, treatment duration, and assessed substances (saffron and crocin). Another important factor could be the concurrent intake of different anti-inflammatory drugs, which might interact with the effects of the tested substance. In general, we need more human studies to solve this paradox.

***Pharmacokinetic***

In most clinical trials saffron and its active component, crocin, were orally administered. It has been reported that different types of crocin, whose differences originated from their glycosyl groups (glucosyl or gentobiosyl), turn into crocetin during the intestinal absorption by hydrolysis of glycosyl groups. This process seems to be specific to the gastrointestinal pathway because intravenous injection of crocin does not cause a high level of crocetin. It is also indicated that crocetin has rapid absorption and a low albumin binding affinity, so it distributes easily to the tissues and also to the central nervous system. Animal studies have shown that crocetin is mainly metabolized in the liver and intestine by glucuronidation. It is also excreted via feces in the animals (150). Some aspects of saffron pharmacokinetics are yet to be determined in humans.

***Adverse effects and safety***

Based on many previous clinical trials, particularly in the field of neurological disorders, 30 mg/day of saffron for one month to 12 months did not induce any significant side effects in patients; however, some tolerable side effects have been reported with low incidence. More common side effects among different studies were gastrointestinal discomfort, fatigue, dizziness, headache, dry mouth, decreased or increased appetite, sweating, anxiety, tremor, and some rare ones were palpitation, hypomania, menometrorrhagia, dyspnea, and bleeding gums. These adverse effects appeared in the patients, not healthy individuals (24, 25, 28, 36-38, 40-42, 44, 47, 50, 52, 53). A double-blind and placebo-controlled trial on 30 healthy volunteers has been conducted to assay possible side effects of 200 and 400 mg/day of saffron for one week. The findings demonstrated that 400 mg/day of saffron significantly decreased the average systolic blood pressure, hemoglobin, and red blood cell count and increased the serum concentration of sodium ion, blood urea nitrogen (BUN), and creatinine in comparison with the placebo. However, these changes were in the normal range (140). Another similar study (randomized, double-blind, and placebo-controlled) on 20 mg/day of crocin for one month revealed that it did not develop major side effects and only reduced amylase, mixed white blood cells, and partial thromboplastin time (PTT) (151). The clinical trials which have been explained so far have applied a dose range of 5–1500 mg/day of saffron which was totally and relatively safe. Toxicological data about saffron suggest that doses up to 1.5 g/day are safe and higher doses than 5 g/day can lead to toxic effects. Besides, It can cause abortion in doses higher than 10 g/day and lethal side effects in doses higher than 20 g/day (26). In an article, different safety studies on animals and humans were reviewed and it concluded that pharmacological doses of saffron are safe, and safranal in high doses is more toxic than total extract or crocin (152). 

**Table 1:** Some details of saffron treatment and study design against depressive disorders in existing clinical trials

Study reference	Assessed effects	Treatments	Duration of treatment	Sample size (final)
(36)	Mild-to-moderate MDD	Group-1: saffron extract: 30 mg/day (TID) Group-2: Imipramin: 100 mg/day (TID)	6 weeks	30
(37)	Mild-to-moderate MDD	Group-1: saffron extract: 30 mg/day (BID) Group-2: Fluoxetine: 20 mg/day (BID)	6 weeks	40
(38)	Mild-to-moderate MDD	Group-1: Saffron extract: 30 mg/kg (BID) Group-2: Placebo (BID)	6 weeks	40
(40)	Mild-to-moderate MDD	Group-1: Petal extract of *C. Sativus*: 30 mg/day Group-2: Placebo	6 weeks	36
(41)	MDD	Group-1: Petal extract of *C. Sativus*: 30 mg/day (BID) Group-2: Fluoxetine: 20 mg/day (BID)	8 weeks	40
(42)	Mild-to-moderate MDD	Group-1: Saffron (stigmas extract): 30 mg/day (BID) Group-2: Petal extract of *C. Sativus*: 30 mg/day (BID)	6 weeks	44
(47)	Mild-to-moderate MDD	Group-1: Crocin 30 mg/day (BID) + (fluoxetine 20 mg/day or sertraline 50 mg/day or citalopram 20 mg/day) Group-2: Placebo + (fluoxetine 20 mg/day or sertraline 50 mg/day or citalopram 20 mg/day)	4 weeks	40
(48)	Depression and sexual desire in CAD patients	Group-1: Saffron: 30 mg/day Group-2: Crocin: 30 mg/day Group-3: Placebo	8 weeks	58
(49)	Depression in patients with MetS	Group-1: Crocin: 30 mg/day (BID) Group-2: Placebo (BID)	8 weeks	33
(44)	Mild-to-moderate MDD	Group-1: Saffron 80 mg/day + Fluoxetine 30 mg/day (BID) Group-2: Saffron: 40 mg/day + Fluoxetine 30 mg/day (BID)	6 weeks	54
(45)	MDD	Group-1: Saffron powder 30 mg/day + Fluoxetine 20 mg/day Group-2: Placebo + Fluoxetine 20 mg/day	4 weeks	30
(39)	MDD in old individuals	Group-1: Saffron: 60 mg/day, in the morning Group-2: Sertraline: 100 mg/day	6 weeks	46
(46)	Persistent Depression	Group-1: Affron^® : ^28 mg/kg (BID) + a prescribed anti-depressant Group-2: Placebo (BID) + a prescribed anti-depressant	8 weeks	139
(50)	MDD in the patients with PCI	Group-1: Saffron extract: maximum 30 mg/day Group-2: Fluoxetine: maximum 40 mg/day	6 weeks	40
(52)	Mild-to-Moderate Postpartum Depression	Group-1: Saffron: 30 mg/day (BID) Group-2: Placebo (BID)	8 weeks	56
(53)	Mild-to-Moderate Postpartum Depression	Group-1: Saffron: 30 mg/day (BID) Group-2: Fluoxetine: 40 mg/day (BID)	6 weeks	64
(54)	Mild-to-Moderate Postmenopausal Depression	Group-1: Saffron: 30 mg/day (BID) Group-2: Placebo (BID)	6 weeks	56
(58)	Depression and Anxiety	Group-1: Saffron stigmas: 100 mg/day (BID) Group-2: Placebo (BID)	12 weeks	54
(59)	Depression and Anxiety	Group-1: Saffron: 30 mg/day (BID) Group-2: Citalopram: 40 mg/day (BID)	6 weeks	60
(60)	Depression and anxiety in diabetic patients	Group-1: Saffron: 30 mg/day (BID) Group-2: Placebo (BID)	8 weeks	54
(61)	Mild-to-Moderate Anxiety or Depression in young cases	Group-1: Saffron: 28 mg/day (BID) Group-2: Placebo (BID)	8 weeks	68
(62)	Self-perceived decreased mood	Group-1: Saffron: 28 mg/day Group-2: Saffron: 22 mg/day Group-3: Placebo	4 weeks	121
(30)	Post-CABG Depression and Anxiety	Group-1: Saffron: 30 mg/day (BID), from 2 days before the surgery Group-2: Placebo (BID)	12 weeks	37
(66)	Anxiety or Sleep Disorder in diabetic patients	Group-1: Saffron: 300 mg/day Group-2: Placebo	One week	30

* MDD:Major Depressive Disorder; TID: Divided into three doses per day; BID: Divided into two doses per day; CAD: Coronary Artery Disease; Mets: Metabolic Syndrome; PCI: Post-Percutaneous Coronary Intervention; CABG: Coronary Artery Bypass Graft

**Table 2:** Summary of clinical trials related to the saffron effects

Disease	Dose range of saffron or its active ingredients	Efficacy in brief	References
Cognitive disorders	30 mg/day	Controversial effects	(24-32)
Depression	30 mg/day	Significant effects	(36-42, 44-50, 52-54, 153)
Comorbid depression/anxiety	28-100 mg/day	Significant effects	(30, 58-62, 66)
PMS	30 mg/day	Significant effects	(75)
OCD	30 mg/day	Relatively significant effects	(77)
ADHD	weight<30 kg, 20 mg/kg weight >30 kg, 30 mg/day	Relatively significant effects	(80)
Pain	300-1500 mg/day	Significant effects	(85, 86)
Sleep disorder	15–28 mg/day	Relatively significant effects	(81, 83, 84)
Cerebral ischemic stroke	200 mg/day	Relatively significant effects	(88)
Metabolic syndrome	30–100 mg/day	Controversial effects	(94-100)
Diabetes	30–1000 mg/day	Controversial effects	(102-105)
Obesity	30–176.5 mg/day	Controversial effects	(108-110)
Fatty liver disease	100 mg/day	Controversial effects	(111)
AMD	20–50 mg/day	Significant effects	(116-121)
POAG	30 mg/day	Boosting effects	(123)
DM	Crocin: 5 and 15 mg/day	Boosting effects with higher dose	(124)
Sexual dysfunction	Oral: 30–200 mg/day Topical gel 1%	Controversial effects	(125-129)
Infertility	60 mg/every other day, 60 mg/day	No significant effects	(130, 131)
Allergic asthma	100 mg/day	Significant effects	(146, 147)
Atherosclerosis	100 mg/day	Relatively significant effects	(132-134)
HTN	1 g saffron stewed for 10 min	Relatively significant effects	(140, 141)
IBS	30 mg/day	Relatively significant effects	(144)
Arthritis/Osteoarthritis	Saffron:100 mg/day Crocin:15 mg/day	Relatively significant effects	(148, 149)

*PMS, Premenstrual Syndrome; OCD, Obsessive-Compulsive Disorder; ADHD, Attention Deficit Hyperactivity Disorder; AMD, Age-Related Macular Degeneration; POAG, Primary Open-Angle Glaucoma; DM, Diabetic Maculopathy; HTN, Hypertension; IBS, Irritable Bowel Syndrome.

## Conclusion

Saffron and its active ingredients exert clinically significant protective effects against various types of depression, age-related macular degeneration, and allergic asthma. In some cases such as cognitive disorders, metabolic disorders, and sexual dysfunction, saffron effectiveness is clinically open to dispute or there are conflictive data about its positive influences; thus, it needs more clinical trials or meta-analyses to be confirmed (Table 2). The dose range of the clinical administration of saffron was 30-100 mg/day usually during chronic or sub-chronic evaluations. Safety assessments revealed that this extract with this dose range is generally safe for humans. However, saffron should not be used by pregnant women because of the limited clinical evidence during pregnancy. 

## Authors’ Contributions

HH recommended the review topic, supervised, directed, managed the study, and approved the final version to be published ; SFO collected the articles, processed data, and prepared the draft of the manuscript.

## Conflicts of Interest

The authors declare no conflicts of interest.
